# The role of bipolar disorder and family wealth in choosing creative occupations

**DOI:** 10.1038/s41598-024-61320-y

**Published:** 2024-05-10

**Authors:** Barbara Biasi, Michael S. Dahl, Petra Moser

**Affiliations:** 1 Yale School of Management, New Haven, USA; 2 National Bureau of Economic Research, Cambridge, USA; 3 Aalborg University, Aalborg, Denmark; 4 NHH Norwegian School of Economics, Bergen, Norway; 5 Aarhus University, Aarhus, Denmark; 6 New York University Stern School of Business, New York, USA

**Keywords:** Mental health, Creativity, Occupational choice, Parental wealth, Bipolar disorder, Human behaviour, Risk factors

## Abstract

Research in psychology and medicine has linked mental health disorders, and particularly bipolar disorder (BD), to employment in creative professions. Little is known, however, about the mechanisms for this link, which could be due to biology (primarily through a person’s genes) or environmental (through socioeconomic status). Using administrative data on mental health diagnoses and occupations for the population of Denmark, we find that people with BD are more likely to be musicians than the population, but less likely to hold other creative jobs. Yet, we also show that healthy siblings of people with BD are significantly more likely to work in creative professions. Notably, people from wealthy families are consistently more likely to work in creative professions, and access to family wealth amplifies the likelihood that siblings of people with BD pursue creative occupations. Nevertheless, family wealth explains only a small share of the correlation between BD and creative employment.

Research in psychology and medicine has pointed to a link between mental health disorders and creative pursuits, such as employment in creative professions. For instance, studies of Swedish population data have found that writers (but not people in other creative professions) face an elevated risk of bipolar disorder (BD), schizophrenia, and depression^1,2^. Similarly, polygenic risk scores for individuals in Iceland indicate that people with an elevated genetic predisposition for BD and schizophrenia are more likely to work in creative professions^3^. [Polygenic risk scores measure a person’s genetic predisposition for a trait or disorder, abstracting from environmental factors (https://www.genome.gov/Health/Genomics-and-Medicine/Polygenic-risk-scores).]

Despite this evidence, less is known about the mechanisms that link creativity with mental health. One set of possible causes is neurobiological. Existing studies point to the importance of dopamine, a neurotransmitter that regulates our perception of pleasure and the ability to think and plan. Dopamine regulation is affected in people with mood disorders, such as BD ^4^. At the same time, dopamine is related to divergent thinking^3,5^, which allows for greater freedom to pursue high-risk projects and fresh ideas that are essential for creative work^6,7^.

Another set of possible causes is environmental. There could be aspects of a person’s background—such as socioeconomic status—which influence both their likelihood of being in a creative profession and their mental health. For example children from families with income in the top percentile have been shown to be 10 times as likely to become inventors as those from below-median income families^8^. Similarly,^9^ have emphasized the importance of exposure to role models and parental income^9^. Conversely, financial distress has been shown to negatively impact mental health^10,11^ and increase the incidence of BD^12–14^ and other mental health disorders. Access to specialized mental health care has also been shown to depend, at least in part, on a person’s socio-economic status^9,15^.

Connecting these insights from psychology and economics, we study the channels behind the observed link between mental health and creativity, with a focus on the role of socioeconomic factors such as parental wealth. We focus on BD, a prevalent “brain disorder that causes unusual shifts in mood, energy, activity levels, and the ability to carry out day-to-day tasks.”[https://www.nimh.nih.gov/health/topics/bipolar-disorder/index.shtml, accessed November 22, 2019.] BD affects 1 in 11 people in the US population and 40 million people world-wide, creating major career costs for affected individuals^16,17^ BD, however, has also been strongly and consistently associated with creative employment^2,3,18^. For instance, biographical evidence suggests that many exceptionally creative individuals were affected by BD, including visual artists such as Vincent van Gogh, writers such as Virginia Woolf, and composers such as Robert Schumann^18^. Our goal is to investigate this tension and explore a potential role of familial wealth in the connection between creativity and BD.

To perform this analysis, we use registry data on mental health diagnoses, creative employment, and parental wealth for the population of Denmark. These data include individual-level administrative records of mental health diagnoses and occupations for all 2,524,325 people who were active in Denmark’s labor force between 1995 and 2015. Family identifiers, available for 71 percent of the population, allow us to identify siblings of people with a mental health condition and observe family wealth. To define employment in creative professions, we implement definitions from psychology which include designers, university teachers (academics), visual artists, architects, display artists, performing artists, musicians, and photographers^2,3,19^.

We begin by revisiting the association between mental health and creativity. We compare the likelihood of creative employment among people with BD, their siblings, and the population. We find that while people with BD are 20 percent *less* likely to be active in any creative occupation than the population, they are 50 percent more likely to be composers and musicians. Occupations that people with BD are *most* likely to pursue, instead, include clerks, librarians, archivists and curators, as well as waiters and bartenders.

Notably, we also find that healthy siblings of people with BD are 11 percent more likely to work in creative professions, confirming the results for Sweden^1^. This finding is consistent with a biological explanation for the link between mental health and creativity. Siblings of people with BD may be affected by a milder (subthreshold) form of BD that eludes diagnosis and experience a greater penchant for divergent thinking, without suffering the adverse health effects of BD, which could lead them to successfully pursue creative employment^20^.

An increased likelihood of creative employment for siblings of people with BD, however, is also consistent with an alternative, socioeconomic explanation: people who grew up in wealthy families have better access to both medical care (including diagnoses for mental health disorders) and employment in creative professions (for instance, because they can afford specialized training required for these jobs). To investigate this alternative channel, we first examine whether people from wealthy families are more likely to hold creative jobs. Then, we test whether the link between mental health and creativity can be explained by differences in parental wealth.

Our results indicate a strong link between parental wealth and employment in creative professions. People from the top decile of the parental wealth distribution are 7 times more likely to work in creative occupations compared with people from the bottom decile. These findings extend existing results for patentees^1,2^ to a broader set of creative occupations.

Nevertheless, the correlation between wealth and creative employment explains only a small portion of the correlation between BD and creativity. Differences in parental wealth can account for at most 8 percent of the correlation between creativity and mental health. Notably, the gradient between parental wealth and creative employment is stronger for siblings of people with BD than for the population. This suggests that environmental factors can reinforce the biological channel, even though they cannot fully explain the link between mental health and creativity.

Our paper contributes to two main strands of literature. The first has explored the link between mental health and creativity. The channels behind the BD-creativity link are still still poorly understood. Polygenic risk scores for BD and schizophrenia are correlated with elevated odds of creative employment in a sample of 86,000 people in Iceland^3^. Yet, polygenic risk scores explain less than 1 percent of these odds, suggesting a statistically significant but small role for biology. In Swedish administrative data, people who have been hospitalized with BD have been found to be over-represented in creative professions, while those with depression are not ^1^. In a similar setting, people in creative professions—and particularly writers—are more likely to have BD, but not other psychiatric disorders^2^. Our study complements these works by analyzing the entire population of Denmark and by considering all people with a BD diagnosis, including those with less severe symptoms, not requiring hospitalization. In addition, the richness of our data and a broader classification of creative professions (which combines definitions previously used in the literature) allow us to explore the link between this condition and each creative occupation individually. Lastly, we investigate the role of what is perhaps the most powerful alternative explanation—socioeconomic status, which has been linked to creativity for inventors^1,2^.

Our paper also relates to research on the relationship between health and work, and occupational choice more specifically^21,22^. Most of this research considers occupational choice as a factor influencing health (for example through differences in the intensity of work between manual and white-collar jobs). Our research complements these analyses by investigating the link between a particular dimension of health—mental health—and the way it relates to occupational choice through biology and socioeconomic status.

## BD and creative employment

### People with BD are less likely to work in creative jobs

**Figure 1:**
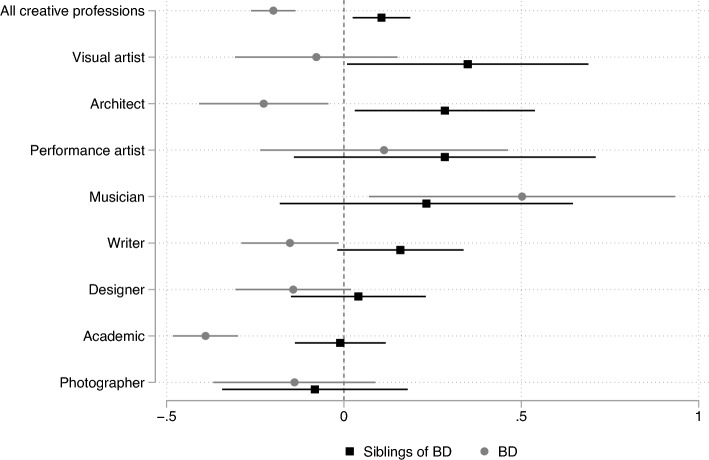
Share in creative occupations: people with BD and their siblings, compared with the population. *Note* OLS estimates (and 95-percent confidence intervals) of *β* in the equation *creative*_*it*_ = *β X*_*i*_ + *γ F*_*i*_ + *θ*_*c(i)*_ + *τ*_*t*_ + *ε*_*it*_ where *creative*_*it*_ equals one if person *i* is employed in a creative profession in year *t*. In the *BD* series, *X*_*i*_ is an indicator for people who have received at least one diagnosis of BD at least once. For *siblings of BD*, *X*_*i*_ indicates siblings of people with *BD*. The variable *F*_*i*_ is an indicator for females. A vector of cohort fixed effects *θ*_*c(i)*_ controls for systematic differences in the propensity to hold a creative job across cohorts; a vector of year fixed effects *τ*_*t*_ controls for differences over time. We report coefficients and confidence intervals as the share of the mean of the dependent variable. Standard errors are clustered at the individual level. Creative professions are listed in Appendix Table A2, together with ISCO-4 codes.

First, we test whether people with BD are more likely to work in creative occupations compared with the population. Figure 1 reports occupation-specific estimates and 95-percent confidence intervals for the difference in the likelihood of holding a creative job between people with BD and the population, controlling for variation across calendar years, birth cohorts, and gender. We divide this difference by the population share of each occupation, so that estimates can be interpreted as percent differences (panel A of Table 1 reports the unscaled differences). These estimates indicate that people with BD are − 0.293 percentage points *less* likely to work in any creative occupation (significant at 1 percent, Table 1, panel A, column 1). Compared with a population share of 1.474 percent, this implies that people with BD are 20 percent less likely to work in a creative occupation (Fig. 1).

**Table 1:** Creative professions and mental health.

	All	Academic	Architect	Designer	Musician	Performance	Photographer	Visual	Writer
	(1)	(2)	(3)	(4)	(5)	(6)	(7)	(8)	(9)
Panel A: BD									
BD	− 2.934***	− 1.891***	− 0.433**	− 0.226*	0.307**	0.046	− 0.141	− 0.049	− 0.553**
	(0.472)	(0.227)	(0.178)	(0132)	(0.135)	(0.087)	(0.117)	(0.074)	(0.254)
Cohort	Yes	Yes	Yes	Yes	Yes	Yes	Yes	Yes	Yes
Year FE	Yes	Yes	Yes	Yes	Yes	Yes	Yes	Yes	Yes
Mean of dep. var	14.742	4.850	1.918	1.586	0.612	0.488	1.012	0.634	3.642
N	48,071,128	48,071,128	48,071,128	48,071,128	48,071,128	48,071,128	48,071,128	48,071,128	48,071,128
Panel B: BD siblings									
BD Sibling	1.560**	− 0.049	0.545**	0.065	0.142	0.139	− 0.083	0.221**	0.580*
	(0.611)	(0.316)	(0.248)	(0.154)	(0.129)	(0.106)	(0.135)	(0.110)	(0.331)
Cohort	Yes	Yes	Yes	Yes	Yes	Yes	Yes	Yes	Yes
Year FE	Yes	Yes	Yes	Yes	Yes	Yes	Yes	Yes	Yes
Mean of dep. var	14.742	4.850	1.918	1.586	0.612	0.488	1.012	0.634	3.642
N	48,071,128	48,071,128	48,071,128	48,071,128	48,071,128	48,071,128	48,071,128	48,071,128	48,071,128

The dependent variable is an indicator for employment in creative professions, defined as in the text, multiplied by 1000. The variable *BD* equals one for individuals with at least one diagnosis of this condition. Each coefficient is estimated from a separate regression. All specifications control for cohort and year fixed effects. Standard errors in parentheses are clustered at the person level. *** denotes coefficients with p-value < 0.1, ** denotes coefficients with p-value < 0.05, and * denotes coefficients with p-value < 0.1.

Occupation-specific estimates reveal moderate heterogeneity in the share of people with BD across occupations. People with BD are 15 percent less likely to be writers, 39 percent less likely to be academics, 23 percent less likely to be architects, and 14 percent less likely to be designers (significant at 5, 1, 5, and 5 percent, respectively, Fig. 1 and Table 1, panel A). People with BD are also 14 percent less likely to be photographers and 8 percent less likely to be visual artists, although these estimates are not statistically significant (with p-values of 0.55 and 0.53, respectively).

Notably, people with BD are 50 percent *more* likely to be musicians and composers (significant at 5 percent). This result is consistent with biographical evidence suggesting that prominent composers (including Berlioz, Brahms, Cherubini, Gluck, Mahler, Mendelssohn, Schubert, and Schumann) may have been affected by bipolar disorder^23^. Interestingly, people with BD are also slightly more likely to be *performing artists* (actors, dancers, choreographers, and directors), though this estimate is smaller and not statistically significant (11 percent, with a p-value of 0.47). [In contrast to our results and those of others^24^, people who are diagnosed with BD in Sweden are more likely to be writers, but not musicians.]

### Healthy siblings of people with BD are more likely to be creative

If BD is associated with creativity through a genetic link, siblings may have a milder “subthreshold” form of BD that allows them to be more creative, without experiencing debilitating symptoms. Models of BD in molecular neuropsychiatry have proposed an inverted U-shaped relationship between the genetic risk for BD and creativity. Greenwood (2016, p. 200)^25^ conjectures that “some aspects of the bipolar spectrum may confer advantages, while more severe expressions of symptoms negatively influence creative accomplishment.”

Our data indicate that siblings of people with BD are 11 percent more likely to work in creative professions compared with the population (Fig. 1 and Table 1, panel B, column 1). Occupation-specific estimates imply that siblings of people with BD are 35 percent more likely to be visual artists, 28 percent more likely to be architects, and 16 percent more likely to be writers (Table 1, panel B, significant at the 5, 5, and 10 percent level, respectively). Siblings are also 23 percent more likely to be musicians and composers than the population, even though this estimate is imprecise due to the small number of observations (with a *p* value of 0.27). These findings confirm the higher share of creative employment among siblings of people who have received in-patient treatment for BD in Sweden^1^.

### Most frequent occupations for people with BD

Looking beyond creative professions, we investigate what type of jobs people with BD are instead most likely to pursue. Formally, we estimate multinomial models of occupational choice, using the broader 3-digit ISCO08 codes to classify occupations and an indicator for BD as the explanatory variable, together with an indicator for women to account for gender differences. [To define occupations consistently over time, we restrict the analysis to 2010–2015, when ISCO08 codes are available. Following the psychology literature, we use 4-digit codes to examine employment in creative professions in the main specifications. To estimate multinomial choice models of an individual’s choice across all occupations, we use 3-digit ISCO codes. An earlier study^26^ estimates a multinomial model of a choice between five occupations, using data from a total of 20,861 interviews in 5 university towns. They find that people with BD and mania are most likely to work in services and show, using a measure of creativity, that services are a creative occupation.]

Multinomial logit estimates also confirm that people with BD are less likely to work in creative professions. For example, people with BD are 34 percent less likely to be “architects, planners, and surveyor designers” and 38 percent less likely to be “artistic, cultural, and culinary associate professionals,” including designers. The largest positive coefficient among creative professions is again for composers, musicians, and performing artists (here included in “creative and performing artists”). People with BD are 27 percent more likely to be employed in this category (significant at 5 percent).

Interestingly, three of the five occupations that people with BD are *least likely* to pursue relate to management. This is in contrast with the popular idea that BD is a “CEO’s disease”^27,28^ because entrepreneurs share certain traits that are associated with BD, including overconfidence and an excessive tolerance for risk. [Medical studies document excessive risk tolerance and impulsive behavior in people with BD. In experiments with a balloon analogue risk task (BART) people with BD score higher on self-reported tests of impulsiveness^29^. Overconfidence and tolerance for risk are also consistent with narcissistic personality^30^. Impulsivity—the tendency to pursue rewards without considering negative consequences—has also been shown to be elevated in people who experience mania^31^.] Rejecting the hypothesis that BD is a CEO’s disease, we find that people with BD are 82 percent *less likely* to be sales, marketing, and development managers, 81 percent less likely to be construction and distribution managers, and 80 percent less likely to be business and administration managers.

**Figure 2:**
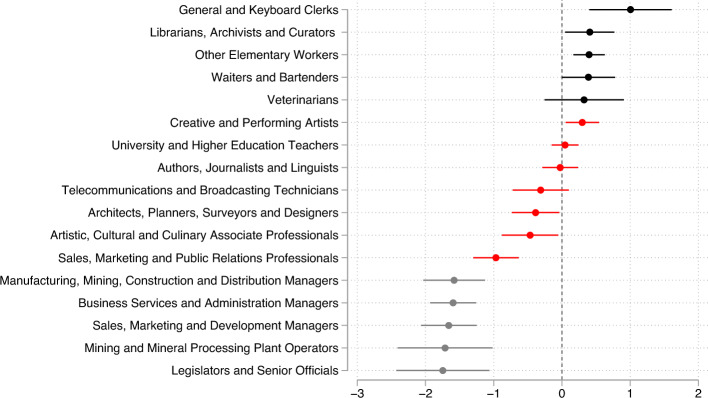
Multinomial logit estimates of occupation: people with BD vs. population. *Note* Multinomial logit estimates and 95 percent confidence intervals of the parameters *β*_*j*_ in equation *Pr(Y*_*it*_ = *j)* = *exp(β*_*j*_* BD*_*i* +_ *θ*_*j*_*F*_*i*_*)/ Σ*_*k*_* exp(β*_*k*_* BD*_*i* +_ *θ*_*k*_*F*_*i*_*),* where *Y*_*it*_ is the occupation of person *i* in year *t*, *BD*_*i*_ equals one for people with BD, and *F*_*i*_ is an indicator for females. This figure shows the five largest and the five smallest estimates of *β*_*j*_, along with estimates of *β*_*j*_ for creative occupations, defined by 3-digit ISCO08 codes. Standard errors are clustered at the individual level.

Instead, people with BD are 177 percent more likely to be clerks; 50 percent more likely to be librarians, archivists, and curators; and 43 percent more likely to be waiters and bartenders (Fig. 2).

## Can parental wealth explain the link between BD and creativity?

Our analysis of population data for Denmark indicates a link between mental health and creativity. In this final section we test whether this link can be explained by differences in family backgrounds, and specifically parental wealth.

### Children of wealthier parents are more likely to be employed in creative professions

We first test whether the finding that people with wealthy parents are more likely to become inventors extends to other creative occupations. Specifically, we plot the share (and 95-percent confidence interval) of people employed in creative professions by their decile of parental wealth.

**Figure 3:**
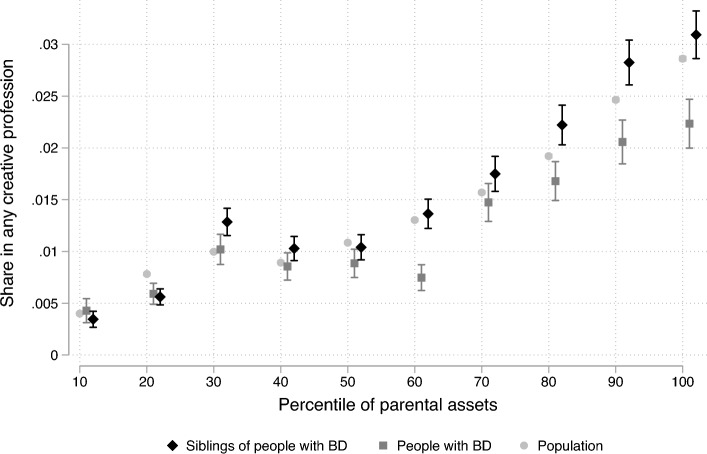
Creative professions and parental wealth: population, people with BD, and their siblings. *Note* Share of people employed in any of the eight creative professions in Fig. 2, separately by the median decile of parental asset for people with BD, their siblings, and the population (with 95-percent confidence intervals). Implementing definitions from psychology, we define creative professions to include academics, architects, designers and display artists, musicians, performance artists, photographers, visual artists, and writers (Appendix Table A2).

We find that people in the top decile of parental wealth are 7 times more likely to work in creative professions (with 2.9 percent, Fig. 3) compared with people in the bottom decile (just 0.4 percent). This suggests that earlier findings based on inventors (a profession where financial resources are needed to be able to patent^1,2^) hold more broadly across creative professions: differences in parental income and wealth help shape the link between innate creativity and professional outcomes.

### Employment in creative professions for people with BD and their siblings

Higher rates of creative employment for healthy siblings are consistent with a biological link between BD and creativity. Yet, analyses of polygenic risk scores have found that only 1.2 percent of the variance in creative employment can be explained by genes that are associated with BD^3^. Indeed, an increased chance of creative employment for BD siblings is also consistent with an environmental explanation for the link between BD and creativity, as siblings share family backgrounds.

To better quantify the role of environmental factors, we examine whether parental wealth can account for the observed relationship between mental health and creative employment. We have shown above that people with wealthier parents are more likely to hold creative jobs. Differences in income and wealth can also directly impact mental health^10,11^. For instance, recipients of large and unconditional cash transfers in rural Kenya experienced significant increases in psychological well-being^10^.

To test this hypothesis, we use information on parental assets and investigate how differences in wealth affect the link between mental health and creativity. We formally test for the influence of parental wealth by re-estimating the difference in the likelihood of holding a creative job between people with BD and the population, controlling for indicators for low (below median) and high (above median) parental wealth.

This exercise indicates that the link between BD and creative employment is robust to controlling for, and cannot be explained by, parental wealth: estimates of the BD-population difference are essentially unchanged when we control for wealth. In these specifications, people with BD are 61 percent more likely to be musicians (Table 2, panel A, column 5), while they are 50 percent more likely when we do not control for wealth (Fig. 3, panel A). Across all occupations, people with BD are 19 percent less likely to have a creative job (Table 2, panel A, column 1, significant at 1 percent), while they are 20 percent less likely not controlling for wealth. These results indicate that only a small share—approximately 6 percent—of the overall association between creativity and mental health can be explained by differences in wealth. All results are robust to alternative definitions of wealth, using of terciles, quartiles or other quantiles of parental assets.

**Table 2:** Creative professions and bipolar disorder.

	All	Academic	Architect	Designer	Musician	Performance	Photographer	Visual	Writer
	(1)	(2)	(3)	(4)	(5)	(6)	(7)	(8)	(9)
Panel A: BD									
High Parental Wealth	8.629***	3.348***	1.196***	0.778***	0.216***	0.106***	0.228***	0.295***	2.462***
	(0.152)	(0.082)	(0.057)	(0.043)	(0.029)	(0.023)	(0.036)	(0.024)	(0.083)
BD	− 2.772***	− 1.566***	− 0.401**	− 0.516***	0.376**	0.060	− 0.027	− 0.107	− 0.590*
	(0.571)	(0.275)	(0.203)	(0.147)	(0.162)	(0.108)	(0.150)	(0.084)	(0.319)
Cohort	Yes	Yes	Yes	Yes	Yes	Yes	Yes	Yes	Yes
Year FE	Yes	Yes	Yes	Yes	Yes	Yes	Yes	Yes	Yes
Mean of dep. var	14.742	4.850	1.918	1.586	0.612	0.488	1.012	0.634	3.642
N	35,353,460	35,353,460	35,353,460	35,353,460	35,353,460	35,353,460	35,353,460	35,353,460	35,353,460
Panel B: BD siblings									
High Parental Wealth	8.645***	3.354***	1.200***	0.780***	0.216***	0.107***	0.227***	0.296***	2.466***
	(0.152)	(0.082)	(0.057)	(0.043)	(0.029)	(0.023)	(0.036)	(0.024)	(0.083)
BD Sibling	1.697***	0.263	0.533**	0.050	0.194	0.130	− 0.112	0.210*	0.428
	(0.611)	(0.316)	(0.249)	(0.154)	(0.129)	(0.106)	(0.135)	(0.110)	(0.331)
Cohort	Yes	Yes	Yes	Yes	Yes	Yes	Yes	Yes	Yes
Year FE	Yes	Yes	Yes	Yes	Yes	Yes	Yes	Yes	Yes
Mean of dep. var	14.742	4.850	1.918	1.586	0.612	0.488	1.012	0.634	3.642
N	35,353,460	35,353,460	35,353,460	35,353,460	35,353,460	35,353,460	35,353,460	35,353,460	35,353,460

The dependent variable equals 1000 for people who are employed in a creative profession, defined by the column titles. *High Parental Wealth* is an indicator for people above the median of parental wealth. The variable *BD* equals one for people who have been diagnosed with BD at least once; the variable *BD Sibling* equals one for siblings of people with *BD.* Standard errors in parentheses are clustered at the individual level. *** denotes coefficients with p-value < 0.01, *** denotes coefficients with p-value < 0.05, and * denotes coefficients with p-value < 0.1.

*OLS* controlling for parental wealth.

Estimates looking at siblings of people with BD paint a similar picture: controlling for parental wealth in Eq. (2) leaves the estimates virtually unchanged. Controlling for wealth, siblings of people with BD are 12 percent more likely to work in any creative profession (with an estimated coefficient for *BD Sibling* equal to 0.00169 and a share of people in creative professions equal to 0.0147, Table 2, panel B, column 1). Compared with estimates that do not control for wealth, this implies that differences in wealth explain 8 percent of the observed association between creativity and BD among siblings.

Interestingly, we find that the gradient between wealth and creative employment is *weaker* for people with BD than for the general population (Fig. 2). Among people with BD, 2.2 percent of those with the wealthiest parents work in creative professions (compared with 2.9 percent in the population). By comparison, only 0.4 percent of people with BD with the least wealthy parents work in creative professions, the same rate as the population.

At the same time, the gradient between wealth and creative employment is slightly stronger for healthy siblings of people with BD than for the population. Among siblings of people with BD, 3.0 percent of those with the wealthiest parents and only 0.4 percent of those with the least wealthy parents work in creative professions. This finding suggests that differences in wealth may amplify the biological links between mental health and creativity.

## Discussion

Using individual-level data on mental health diagnoses and occupations, we revisit the link between creativity and mental health disorders and study the channels behind this relationship, with a specific focus on biological and environmental forces. We show that people with BD are 50 percent more likely to be musicians, but less likely to be employed in other creative professions. Notably, healthy siblings of people with BD are consistently more likely to work in creative professions. For instance, siblings of people with BD are 16 percent more likely to be writers, 28 percent more likely to be architects, and 28 percent more likely to be visual artists. These findings indicate that family-level traits, either through genes or socioeconomic status, link mental health disorders with creativity.

Consistent with a strong influence of socioeconomic status, we find that people with parents in the top quartile of parental wealth are over 7 times as likely to hold a creative job compared with those in the bottom quartile. This striking correlation, however, explains only a small share—no more than 8 percent—of the observed correlation between mental health and creative employment. Interestingly, we also find that the creativity-wealth gradient is stronger for siblings of people with BD than for the population.

Taken together, our findings indicate that neurobiological factors may be the primary link between mental health and creativity, and that socioeconomic status amplifies this biological link. Siblings people with BD from high-income families may be additionally more likely to pursue creative jobs because their occupational choices are less constrained by financial needs, or because high-income people are more likely to experience positive moods. While comprehensive, our analysis faces a few limitations. First, our measure of creativity is limited to the choice of creative occupations and may therefore miss other expressions of creativity that do not result in creative employment. We may be missing expressions of Big Creativity^32,33^, which have been linked to mental health conditions^34^. Empirically, expressions of Big Creativity can be proxied through creative output, e.g., through inventions^8^, publications^31^, or musical creations^37^ future analysis could establish such links by linking health records with these measures. Second, it is possible for the set of occupations that have a creative component to change over time, due to changes in the nature of work. Our time-invariant classification of creative occupations makes it possible to compare our results to findings in previous literature^1,2^ but is not able to explicitly incorporate such changes. Third, our analysis of the relationship between BD, creative employment, and wealth is not causal and there is the possibility of reverse causality. For example, it is possible that BD leads to certain choices of occupation, but it is also possible that the choice of a certain job creates stress, e.g., over financial uncertainty, which may trigger latent pre-dispositions to develop BD and other mood and anxiety disorders^35^.

## Methods

### Data and sample

We use registry data on the population of Denmark, obtained from Statistics Denmark. Our data comprise mental health diagnoses and occupations for 2,524,325 people born between 1946 and 1975. Family identifiers, which we use to link people to their siblings and measure differences in parental wealth, are available for 71 percent of the population. Appendix Table A1 summarizes the variables used in our analysis. While we are not allowed to share the data ourselves, researchers will be able to access it through an application with Statistics Denmark. [https://sundhedsdatastyrelsen.dk/da/english/health_data_and_registers/research_services/apply/data_statistics_dk].

#### Mental health diagnoses

Information on diagnoses is taken from the Central Psychiatric Register (*Landspatientregistret for Psykiatri Diagnoser*), which records all mental health diagnoses in Denmark between January 1, 1995, and December 31, 2015. The Register classifies mental health disorders according to the World Health Organization International Statistical Classification of Diseases and Related Health Problems (ICD-10; see http://apps.who.int/classifications/icd10/browse/2016/en#/F30-F39).

Implementing this classification, our variable *BD* identifies 18,729 people who have received at least one diagnosis of bipolar disorder (ICD-10: F31) or mania (ICD-10: F30). BD is defined as “A disorder characterized by […] some occasions of an elevation of mood and increased energy and activity (hypomania or mania) and on others of a lowering of mood and decreased energy and activity (depression).” Mania is described as “A disorder […] which varies from carefree joviality to almost uncontrollable excitement, […] accompanied by increased energy, resulting in overactivity, pressure of speech, and a decreased need for sleep.”

#### Creative occupations

In this analysis, we measure a person’s tendency for creativity through employment in creativity professions. Existing economic analyses have measured creativity through output, such as patents^8,9^, publications^36^, and musical compositions^37^. By contrast, studies of mental health disorders and creativity in psychology and medicine have treated creativity as an individual-level characteristic, proxied by a person’s choice of occupation^1,2,19^.

We follow the psychology literature in defining creative occupations. Previous studies have classified creative professions to include designers, writers, academics, visual artists, architects, display artists, performing artists, composers, and musicians^19^. Others have excluded architects but included photographers^1^. We include both architects and photographers among the creative occupations and report results separately by occupation.

Denmark’s registries follow the International Standard Classification of Occupations (ISCO) to classify occupations. Years 1995–2009 use the 1988 classification and years 2010–2015 use the 2008 classification. Using 4-digit ISCO codes we distinguish academics (ISCO code 2310), photographers (3131), visual artists (2452 in 1988; 2651 and 2166 in 2008), designers (3471 in 1988; 3432, 3435, 2163, and 2166 in 2008), performing artists (2454 and 2455 in 1988; 2654 and 2655 in 2008), composers and musicians (2453 in 1988, 2652 in 2008), writers (2451 in 1988; 2431, 2432, 2641, and 2642 in 2008), and architects (2141 in 1998; 2161 and 2162 in 2008; Appendix Table A2). In multinomial logit regressions we aggregate ISCO codes to the 3-digit level to reduce the number of choices.

#### Family identifiers and parental wealth

To match people with their siblings, we use their mother’s or father’s social security number as a family identifier. Family identifiers are available for 1,788,166 people (71 percent of the population); 75 percent of them have one or more siblings. Family identifiers allow us to identify siblings of people with BD.

Data on parental wealth are available for people whose mother or father reported assets for at least one year between 1980 and 2015. We set assets to zero for people whose parents are listed but do not have any financial assets. Assets are reported by banks and other financial institutions and not by the individuals themselves. All results are robust to excluding individuals without information on parental assets from the analyses. To define a person’s position in the distribution of parental wealth, we calculate the percentile of parental assets for each year (from 1980 to 2015) and assign each person to their parents’ median percentile across all years.

### Empirical framework

#### BD and creative employment

First, we test whether people with BD are more likely to work in creative occupations compared with the population. To avoid picking up differences in labor force participation between people with BD and the population, we restrict attention to people with positive earnings in any given year. We estimate the following equation separately for eight creative occupations, including writers, academics, architects, designers, musicians, photographers, visual artists, and performing artists:

1 $$creative_{it} = \beta BD_{i} + \gamma F_{i} + \theta_{c\left( i \right)} + \tau_{t} + \varepsilon_{it}$$

where the variable *creative*_*it*_ equals one if person *i* is employed in any or in a specific creative profession in year *t*, and *BD*_*i*_ equals one if the person has been diagnosed with BD at least once. An indicator for women *F*_*i*_ controls for possible gender differences in occupational choices. A vector of cohort fixed effects *θ*_*c(i)*_ controls for systematic differences in the propensity to hold a creative job across cohorts. A vector of year fixed effects *τ*_*t*_ controls for differences in the same propensity over time. We cluster standard errors at the individual level. The coefficient *β* estimates the difference in the likelihood of holding a creative job between people with BD and the population, controlling for variation in creative employment across calendar years, birth cohorts, and gender.

#### Creative employment among siblings of people With BD

If BD is associated with creativity through a genetic link, siblings may have a milder “subthreshold” form of BD that allows them to be more creative, without experiencing debilitating symptoms. Models of BD in molecular neuropsychiatry have proposed an inverted U-shaped relationship between the genetic risk for BD and creativity^25^. Conjectures that “some aspects of the bipolar spectrum may confer advantages, while more severe expressions of symptoms negatively influence creative accomplishment.”

To test whether healthy siblings of people with BD are more likely to pursue creative jobs, we estimate Eq. (1) with an indicator for *BD siblings* instead of the indicator for BD:

2 $$creative_{it} = \beta_{S} BD \, sibling_{i} + \gamma F_{i} + \theta_{c\left( i \right)} + \tau_{t} + \varepsilon_{it}$$

In this modified equation, the coefficient *β*_*S*_ estimates the difference in the likelihood of holding a creative job between siblings of people with BD and other people of the same gender, in the same birth cohort and calendar year.

#### Most frequent occupations for people with BD: multinomial logit

Looking beyond creative professions, we investigate what type of jobs people with BD are instead most likely to pursue. Formally, we estimate multinomial models of occupational choice, using the broader 3-digit ISCO08 codes to classify occupations. To define occupations consistently over time, we restrict the analysis to 2010–2015, when ISCO08 codes are available. Following the psychology literature, we use 4-digit codes to examine employment in creative professions in the main specifications. To estimate multinomial choice models of an individual’s choice across all occupations, we use 3-digit ISCO codes. [An earlier study estimates a multinomial model of a choice between five occupations, using data from a total of 20,861 interviews in 5 university towns. They find that people with BD and mania are most likely to work in services and show, using a measure of creativity, that services are a creative occupation ^28^.]We model the probability that a person works in occupation *j* at time *t* as:

3 $$\Pr \left( {Y_{it} = j} \right) \, = \, \exp \left( {\beta_{j} BD_{i + \gamma J} F_{i} } \right)/\Sigma_{k} \exp \left( {\beta_{k} BD_{i + \gamma k} F_{i} } \right)$$

where *Y*_*it*_ is the occupation of person *i* in year *t* and *F*_*i*_ is an indicator for women. First, we estimate *β*_*j*_ and *γ*_*j*_ for 129 occupations via maximum likelihood. We normalize both parameters to zero for “Primary Schools and Early Childhood Teachers” (ISCO08 code 234), the most common occupation (with 6.8 percent of workers). We then compute the excess probability of occupation* j* for people with BD as exp(*β*_*j*_)− 1.

### Supplementary Information

Supplementary Information.

## Data Availability

We obtained our data from Statistics Denmark. While we are not allowed to share the data ourselves, researchers will be able to access it through an application with Statistics Denmark. [https://sundhedsdatastyrelsen.dk/da/english/health_data_and_registers/research_services/apply/data_statistics_dk].
