# Supplementary material for: The role of bipolar disorder and family wealth in choosing creative occupations
Source: Sci Rep. 2024 May 10;14:10703. doi: 10.1038/s41598-024-61320-y (PMC11087571; doi:10.1038/s41598-024-61320-y)
Supplement: Supplementary file 1 — Supplementary Information. [file 41598_2024_61320_MOESM1_ESM.pdf]

The Role of Bipolar Disorder and Family Wealth in Choosing Creative Occupations

Barbara Biasi, EIEF, Yale and NBER,
Michael S. Dahl, Aalborg University and Aarhus University, and
Petra Moser, NYU, NBER, and CEPR

Online Appendix

Data Appendix

Information on all demographic variables (age, gender, children, parents, employment and occupations) are drawn from a set of registries previously known as the Integrated Database for Labor Market Research (IDA). These registries combine high-accuracy information across more than 150 government registries.

Data on psychiatric patients are drawn from the LPSYDIAG registry. Data on prescriptions come from the LMDB registry.

Information on families, households and demographics are from the BEF, FAIN, FAM, FTDK, FTDM, UDDA and IDAP registries. Data on employment, occupations, unemployment, income and employers are drawn from the IDAN, IDAS, FIRM, IND and AKM registries. Information on start-ups is drawn from the IVPE and IVPS registries.

We link individual-level variables across these datasets using social security numbers (SSN). People born in Demark receive their SSNs at birth. Immigrants and foreign employees are assigned an SSN by the municipal office or the International Citizen Service when they receive a work permit or residence permit.

We define creative occupations using the ISCO variable in the AKS Danish registry data (variables DISCO88 and DISCO08). We link the ISCO-88 and ISCO-08 using the official correspondence table, available at http://www.ilo.org/public/english/bureau/stat/isco/.

TABLE A1 – LIST OF VARIABLES

Variable	Variable name	Definition	Years available	Registry name	Registry
Prescriptions and Diagnoses					
BD		Indicator for individuals with diagnosis code ICD-10: F31	1995-2015	Landspatientregistret for Psykiatri Diagnoser	LPSYDIAG
Mania		Indicator for individuals with diagnosis code ICD-10: F30, and for which BD = 0	1995-2015	Landspatientregistret for Psykiatri Diagnoser	LPSYDIAG
Labor Market Variables					
Creative occupations	ISCO08, ISCO88	Indicator for individuals with occupation (See Table A2)	1995-2015		
Family				Income and Employment	
Mother ID		Individual identifier of mother	1995-2015	Employment	IND, IDAP and IDAN

TABLE A2– CREATIVE PROFESSIONS

Occupation	ISCO-88	ISCO-08	N. People	N. Obs
Academics	2310 University and Higher Education Teachers	2310 University and Higher Education Teachers	46,945	226,113
Photographers	3131 Photographers	3431 Photographers	10,196	47,764
		3521 Broadcasting and Audiovisual Technicians		
Visual artists	2452 Visual artists (Sculptors, Painters and Related Artists)	2651 Visual artists (Sculptors, Painters and Related Artists)	7,902	30,051
		2166 Graphic and Multimedia Designers		
Designers	3471 Decorators and Commercial Designers	3432 Interior Designers and Decorators	19,076	75,156
		3435 Other Artistic and Cultural Associate Professionals		
		2163 Product and Garment Designers		
		2166 Graphic and Multimedia Designers		
		3433 Gallery, Museum and Library Technicians		
		3435 Other Artistic and Cultural Associate Professionals		
Performing artists	2455 Film, Stage and Related Actors and Directors	2654 Film, Stage and Related Directors and Producers	6,608	23,263
		2655 Actors		
	2454 Choreographers and Dancers	2653 Dancers and Choreographers		
	3474 Clowns, Magicians, Acrobats and Related Associate Professionals			
Composers and musicians	2453 Composers, Musicians and Singers	2652 Musicians, Singers and Composers	5,496	29,255

Writers	2451 Authors, Journalists and Other Writers	2431 Advertising and Marketing Professionals	29,789	171,087
		2432 Public Relations Professionals		
		2641 Authors and Related Writers		
		2642 Journalists		
Architects	2141 Architects, Town and Traffic Planners	2161 Building Architects	11,905	90,080
		2162 Landscape Architects		

Note: Definition of creative professions obtained combining those of Kyaga (2011, 2013) and Ludwig (1992).
